# Hashimoto’s thyroiditis and coexisting disorders in correlation with HLA status—an overview

**DOI:** 10.1007/s10354-021-00879-x

**Published:** 2021-09-15

**Authors:** Peter Mikosch, Adrian Aistleitner, Markus Oehrlein, Eva Trifina-Mikosch

**Affiliations:** 1 Teaching Unit, Medizinische Universität Wien/Medical University Vienna, Vienna, Austria; 2 Dept. Internal Medicinie 2, Landesklinikum Mistelbach-Gänserndorf, Liechtensteinstraße 67, 2130 Mistelbach, Austria

**Keywords:** Thyroid, Autoimmune disorders, Diagnostics, Comorbidity, Genetics, Schilddrüse, Autoimmunerkrankungen, Diagnostik, Komorbidität, Genetik

## Abstract

Hashimoto’s thyroiditis (HT), also known as chronic lymphocytic thyroiditis, is a frequent disorder of the thyroid gland caused by autoimmune-trigged lymphocytic infiltration and destruction of the thyroid gland. With the progressive destruction of the organ, the thyroid gland shrinks in size, thus commonly leading to hypothyroidism. Therapy of HT is mainly focused on managing the thyroid dysfunction by oral substitution of L‑thyroxine. However, patients with HT often complain about a broad spectrum of symptoms, with some of them hardly explained by HT itself. Several other disorders are known to be associated with HT. The etiology of HT seems to be multifactorial, including environmental influences such as iodine supply, infections, and stress as triggers of immune modulation. In addition, also a genetic background based on changes of the human leukocyte antigen (HLA) status seems to be evident. The paper will provide an overview of diseases related to HT, including their correlation to certain HLA patterns. This presentation should give a broader view on HT-related disorders and facilitate detailed examination and management of patients with HT.

## Abstract (de)

Die Immunthyreopathie Hashimoto oder Hashimoto-Thyreoiditis (HT), auch chronisch-lymphozytäre Thyreoiditis, stellt eine häufige Erkrankung der Schilddrüse dar, die durch eine autoimmun getriggerte lymphozytäre Infiltration und nachfolgende Zerstörung der Schilddrüse hervorgerufen wird. Mit zunehmender Organzerstörung nimmt die Größe der Schilddrüse ab, die Folge ist eine Schilddrüsenunterfunktion. Die Therapie der HT zielt vorrangig auf die Normalisierung der Schilddrüsenfunktion bei eingetretener Hypothyreose durch orale Gabe von L‑Thyroxin ab. Patienten mit HT schildern jedoch oftmals ein breites Spektrum an Symptomen, die durch die HT selbst nur schlecht erklärbar sind und die auch nach Normalisierung der Schilddrüsenfunktion anhalten. Diverse andere Erkrankungen sind bekanntlich mit HT vergesellschaftet. Die Pathogenese der HT erscheint nach heutigem Wissensstand multifaktoriell zu sein. Jodzufuhr, Infektionen, Stress wurden als Einflussfaktoren identifiziert, die eine Immunmodulation mit Entstehung einer HT hervorrufen können. Ein genetischer Einfluss in Zusammenhang mit dem human leukocyte antigen(HLA)-Status wurde ebenfalls als einer der ursächlichen Faktoren für HT ermittelt. Die vorliegende Arbeit gibt eine Übersicht über Erkrankungen, die mit HT assoziiert sind, sowie deren Korrelation mit dem HLA-Status. Das Ziel ist, eine erweiterte Sichtweise von HT inklusive assoziierter Erkrankungen zu gewinnen, um so eine bessere und umfassendere Betreuung von Patienten mit HT zu ermöglichen.

## Introduction

Hashimoto’s thyroiditis (HT), also known as chronic lymphocytic thyroiditis or Hashimoto’s disease, was first described by the Japanese physician Hakaru Hashimoto in 1912 as an enlarged thyroid with a chronic lymphatic infiltration. This form of HT is also frequently referred to as the hypertrophic or goitrous form of HT. After many years the thyroid typically shrinks in size, leading to a secondary atrophic form of HT. A thyroid gland of normal or reduced size at diagnosis with a lymphatic infiltration is called Ord thyroiditis. However, in clinical practice, these different forms of HT are usually summarized under the term Hashimoto’s thyroiditis (HT). If not otherwise mentioned, this article will in general refer to both forms of HT (hypertrophic and atrophic), as in most papers the different forms of HT are considered as one disease with different presentations in thyroid sized?>

The frequency of HT is increasing among Caucasians and its prevalence in the population is estimated at approximately 5% [1]. HT is caused by autoimmune-trigged lymphocytic infiltration and destruction of the thyroid gland. Excessively stimulated T CD4+ cells and their differentiated cells (ThH1, Th2, Th17, Treg) and different proinflammatory cytokines such as interferon and interleukin (IL)-17 play an important role in the pathogenesis of HT [1, 2]. The underlying background of the pathological autoimmune reaction towards the thyroid gland is regarded as being multifactorial [3], with environmental influences such as the extent of iodine supply, infections, and stress as triggers of immune modulation [2, 4–6]. Also, the gut microbiota has been discussed as a further possible cause of influencing thyroid immunity and the development of HT [7]. Different studies have shown a changed intestinal microbiota composition in patients with HT as compared to healthy controls, thus possibly having an influence on the reactivity of the immune system and the development of HT [8–11]. However, further studies on this topic have to be awaited to determine whether gut dysbiosis may have an impact on the development of HT [7, 8, 12]. In addition, genetic factors in association with the human leucozyte antigen (HLA) gene, different immunoregulatory genes (*CD25, CD40, FOXP3, CTLA4, PTPN22*), and thyroid-specific genes (thyroid stimulating hormone receptor, thyroglobulin) also play a key role.

With the progressive destruction of the organ by activated macrophages and cytotoxic lymphocytes, the thyroid gland shrinks in size, thus commonly leading to hypothyroidism in the long term. Consequently, therapy of HT is mainly focused on managing the thyroid dysfunction by oral substitution of L‑thyroxine. However, patients with HT often complain about a broad spectrum of symptoms, with some of them hardly explained solely by HT itself. Several other disorders are frequently seen with HT, assuming an association with this thyroid disorder. In clinical practice this necessitates a broad view on HT itself and the spectrum of other disorders related to HT, in order to diagnose and manage all disease aspects.

This paper will provide an overview of diseases related to HT, including their correlation to certain HLA patterns. With this presentation, a broader view on HT and its related disorders will be presented, facilitating detailed examination and management of patients with HT and its associated disorders.

## Hashimoto thyroiditis and genetics

Although the background and cause of HT is multifactorial [3], including environmental influences such as iodine supply, infections, and stress as triggers of immune modulation [2, 4, 6], it became evident that HT is also based on genetic changes and the HLA status. Already in 1978 was *HLA-DRw3* suspected to be more frequent in HT [13], and in 1981, Farid et al. [14] found a higher occurrence of *HLA-DR5* in goitrous thyroiditis, whereas *HLA-DR3* was more frequent in atrophic thyroiditis. Tandon et al. [15] could confirm the association of HT with *HLA-DR3*, but a difference between *HLA-DR5* and *HLA-DR3* in their association to goitrous or atrophic HT variations could not be seen in their study. In 2001, the HLA haplotype DRB1*04-DQB1*0301 was found to increase the risk of developing HT [16]. Another potential connection to HT was seen with genetic changes at the *HLA-DR *pocket, in particular the exchange of the neutral amino acids Ala or Gln for arginine at position beta 74 [17]. In contrast, the association of HT with the *HLA-DR3* family could not be supported in another study from 2002 [18].

There are also other genetic aberrations which may increase the risk for HT. The *CTLA4* gene plays a role in T‑cell activation and is located on chromosome 2q33. In a meta-analysis [19] it could be demonstrated that two frequent single nucleotide polymorphisms (SNPs) of the cytotoxic T-lymphocyte-associated protein 4 (*CTLA4*) gene (+49A/G and CT 60) are associated with HT. Additional sequential analysis showed that the +49A/G polymorphisms may be candidate biomarkers for HT. The risk for HT with +49A/G was higher in the subgroup of Asians as compared to Caucasians. The CT 60 showed no significant association in the Caucasian subgroup [19]. The Forkhead box P3 (*FoxP3*) gene plays a relevant role for regulatory T‑cells concerning development and function. The -2383CC polymorphism of *FoxP3* was found more frequent in severe HT forms than in less severe ones [20]. The *ZFAT* ex9b-SNP10 polymorphism, especially the TT genotype and T allele, showed a significant higher frequency in patients with severe HT compared with less severe HT patients (*p* = 0.0029 and *p* = 0.0049, respectively) [21].

To date, the controversial discussion about the associations of HT with *HLA-DR* and many other gene aberrations is still ongoing. HT is considered a multifactorial disease caused not only by genetic predisposition, but also by other different non-genetic factors, which all increase the risk of developing HT [3].

## Materials and methods

**Fig. 1:**
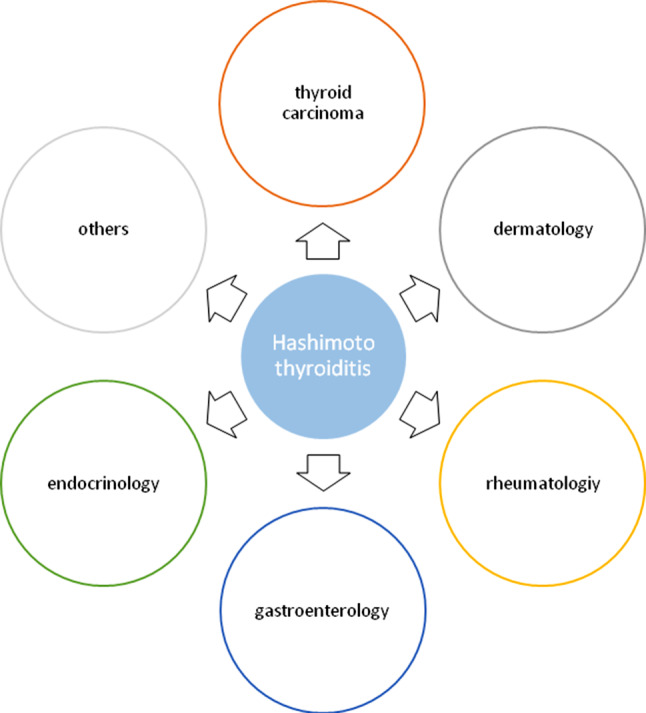
Hashimoto thyroiditis and associated disorders grouped by organ systems

The paper provides a summary of diseases related to HT. The associated disorders will be elucidated from two different points of view. Firstly, as pointed out above, HT is associated with polymorphisms in the *HLA-DR* gene locus [3]. Several other diseases are also associated with changes in the *HLA-DR* gene locus, which thus makes a simultaneous occurrence of these disorders possible. For this, the *HLA-DR* gene was searched in PubMed in terms of its coexistence with HT. Secondly, disorders known to be associated with HT were searched in PubMed and evaluated from a clinical point of view as well. The associated diseases were grouped according to the affected organ systems (Fig. 1).

### Thyroid carcinoma

#### Papillary thyroid carcinoma

Papillary thyroid carcinoma (PTC) is the most frequent form of thyroid carcinoma. Its incidence has been estimated at between 0 and 0.29 per 100,000 (USA) [22]. Genetic aberrations found in the area of the *HLA-DR* gene locus show correlations between the *HLA-DR* gene locus and PTC, similar to HT. Porto et al. [23] compared the HLA status of 315 healthy persons and patients with thyroid pathologies (PTC *n* = 181, follicular carcinoma *n* = 31, lymphocytic thyroiditis *n* = 29, multinodular goiter *n* = 50). Based on their results, the authors considered *DR8* and *DQ4* as independent markers for PTC in Caucasian patients [23]. In another study [24] based on an Iranian population, it was suggested that HLA-DRB1*04 is a predisposing factor for PTC.

Undoubtedly, several studies could show that malignant changes are more likely to occur in connection with HT. The inflammatory process in HT can be seen as a potential risk factor to promote development of thyroid carcinoma [25]. In a single-center study [26], 8524 patients with thyroidectomy were analyzed, of whom 1735 were diagnosed with PTC and 839 with HT. A significantly higher incidence of PTC was found in patients with HT (29.4%) as compared to those without HT (19.4%). Moreover, male HT patients had a significantly higher rate of PTC (44.3%) than female patients (28.3%). Patients with both HT and PTC were younger (43.1 vs. 46.6 years), had smaller nodules (1.10 vs. 1.34 cm), less external invasion (0.4 vs. 2.5%), less lymph node metastasis in the lateral neck area (17.2 vs. 26.9%), and less advanced TNM stages than PTC patients without HT [26].

A respective cohort study [27] showed an increased prevalence of PTC among patients with HT. 7545 patients with thyroidectomy were examined. PTC was more frequent in patients with HT (106/452; 23.5%) vs. in non-HT patients (530/7093; 7.5%) [27].

Another study [28] of 2478 patients who had undergone thyroidectomy also showed a connection between HT and PTC. Patients with PTC presented with a significantly higher prevalence of HT as compared to patients with benign thyroid nodular disease (18.8% vs. 7.2%). Similar to the results of Zhang et al. [26], patients with PTC and HT were younger, showed a female predominance, and had smaller tumors with less advanced TNM stages in comparison to patients without HT [28]. Further significant differences were observed in mean TSH concentrations (2.02 ± 1.76 vs. 1.46 ± 1.21 mU/L), positive thyroglobulin antibodies (TgAb; 40.0% vs. 20.4%), and thyroid peroxidase antibodies (TPOAb; 24.8% vs. 12.5%). In PTC patients with HT, the thyroid-stimulating hormone (TSH) was significantly higher than in patients without HT (2.54 ± 2.06 vs. 1.90 ± 1.66 mU/L). The presence of HT, a higher TSH concentration, male sex, and positive TgAb were shown to be independent risk factors for the development of PTC in multivariate analysis [28].

A review on the topic [29] could also find a correlation between HT and PTC, but found relevant differences between studies based on diagnosing PTC by fine-needle aspiration cytology and others based on histology after thyroidectomy. In eight fine-needle aspiration studies with a total of 18,023 specimens, PTC showed a prevalence of 1.20% in patients with HT, whereas in eight thyroidectomy studies of 9884 specimens, it was 27.56% [29].

Another meta-analysis [30] based on 38 studies including 10,648 PTC cases identified histologically proven HT in 2471 (23.2%) PTCs. HT was more frequent in PTCs than in other types of thyroid carcinomas or benign thyroid diseases. PTCs with coexisting HT were significantly related to female patients (OR [odds ratio] = 2.7), multifocal involvement (OR = 1.5), no extrathyroidal extension (OR = 1.3), and no lymph node metastasis (OR = 1.3). Moreover, PTCs with HT showed a better long-term prognosis with a significant association with long recurrence-free survival (HR = 0.6) [30].

### Dermatology

#### Vitiligo

Vitiligo is characterized as a chronic skin disease with pale patchy depigmented areas of skin due to the loss or a malfunction of melanocytes. Although the pathogenesis behind is not yet fully understood, studies strongly imply that autoimmunity plays a dominant role, next to genetic and environmental factors [31].

Findings on HLA with vitiligo gave evidence of an association with HLA. A study [32] in Chinese Han and Uygur populations (1117 cases with vitiligo and 1429 controls) identified two independent association signals within the MCH regions (rs11966200 and rs9468925). A small study in the Turkish population (41 patients with vitiligo and 61 healthy controls) suggested DRB1*03, DRB1*04, and DRB1*07 alleles as genetic markers for increased rate on developing vitiligo [33].

In a large-scale nationwide population-based retrospective study from Taiwan (14,883 vitiligo patients, 59,532 controls) a significant association between vitiligo and other autoimmune diseases including HT could be confirmed [34]. Daneshpazhooh et al. [35] detected in their study (94 patients with vitiligo, 96 patients without vitiligo) TPOAb at 18.1% in vitiligo-affected patients versus 7.3% in vitiligo-free controls. According to this study, TPOAb was significantly (*p* < 0.025) more common in vitiligo patients as compared to controls, especially in young women [35]. Two other studies assessed the link between vitiligo and thyroid disease in children and adolescents, showing that HT in patients with vitiligo is 1.7–2.5 times more frequent than in the control group. Like others [35], they also detected that vitiligo usually appears before the development of thyroid diseases [36, 37]. Consequently, as vitiligo frequently precedes thyroid dysfunction [35–37], regular screening for thyroid dysfunction and thyroid autoantibodies in patients suffering from vitiligo seems reasonable in order to detect associated thyroid dysfunction at an early timepoint.

#### Urticaria

Urticaria, commonly known as hives, is a dermal efflorescence marked by pale red, raised, itchy bumps and a burning sensation. The etiology of chronic urticaria (CU; hives lasting longer than 6 weeks) is unknown in about 75% of cases and is hence called chronic idiopathic urticaria (CIU) [38]. Out of these, probably more than 50% have an autoimmune background [39]. Acute urticaria (hives lasting less than 6 weeks) is, in most cases, caused by an allergic trigger. Thus, the following section will only refer to CU.

There are only few studies which investigated the HLA status in patients with CU. One was performed with 42 CU patients matched with 193 healthy individuals. All participants were individuals of the Chinese Han population. This study revealed that CU patients had a significant gene frequency increase of HLA-DRB1*12, *0901 and a decrease of HLA-DQB1*05 genes [40]. A further study analyzed 115 patients with CU and 162 healthy controls. HLA-DRB1*04 was significantly more frequent in patients, especially with the autoimmunological subtype of CU [41]. However, a study with 42 Brazilian CU patients showed no significant correlation to any major histocompatibility complex of classes I and II [42].

Several studies examined the association between CIU and thyroid autoimmunity. However, many of them had rather small sample sizes, thus generating low power. Nevertheless, some studies with adequately large study populations presented a significantly higher prevalence of thyroid antibodies and/or HT in patients with urticaria as compared to controls [39, 43].

The meta-analysis of Pan et al. [43] showed that patients with urticaria had a higher prevalence of thyroid autoantibodies as compared to non-urticaria controls (TgAb OR 6.55; thyroid microsomal antibody [TmAb] OR 4.51; TPOAb OR 8.71) [43]. Confino-Cohen et al. [39] collected data of over 12,000 patients with urticaria, with 9.8% of them having hypothyroidism as compared to 0.6% in the control group. Hypothyroidism was more present in women. In female patients with CIU, the probability of having hypothyroidism was 23.07 times greater as compared to controls. On the other hand, male patients with CIU revealed an OR of 7.57 for having hypothyroidism comparing to the control group without CIU [39]. In a case–control study by Nuzzo et al. [44], the prevalence of thyroid antibodies in patients with CIU was more than three times higher compared to controls (22 vs. 6.5%). HT was also more frequent in CIU patients (18.5 vs. 1.8%) [44].

The clinical results show that patients with CIU are more likely to have thyroid autoimmunity. A screening of thyroid function may be useful in all patients with CIU.

#### Alopecia areata

Alopecia areata (AA) has long been associated with thyroid diseases [45]. A meta-analysis based on 3256 cases and 7543 controls showed an association of AA to several HLA loci. According to the study, the HLA-DR antigen is a key region in the development of AA [46].

One small study with only 71 patients investigated AA with its associated diseases. The most frequent co-disorders were thyroid diseases, but the study made no division to specific diseases of the thyroid gland. According to the study, 18.3% out of 71 patients had thyroid diseases (*p* < 0.01) [47].

In a recent large study [45], the association between AA and thyroid diseases was investigated. Two analyses were performed, the first included 5929 AA patients and 59,290 matched controls to evaluate the risk of thyroid diseases. The second analysis included 35,071 patients with thyrotoxicosis, 19,227 patients with Graves’ disease, 5460 patients with thyroiditis, 3352 patients with HT, and matched controls in order to assess the risk of AA in these patients with thyroid disorders. After adjustment of confounding factors, AA patients had an increased risk for all thyroid diseases. On the contrary, patients with thyrotoxicosis, Graves’ disease, and thyroiditis had a significantly increased risk of developing AA, but not patients with HT. Based on these results, a bidirectional association between AA and thyroid diseases seems to be present, with common biological mechanisms underlying these two disorders [45]. Routine thyroid function screening has been recently recommended for all AA patients with a positive family history of thyroid disease, clinical findings of goiter, a history of atopy, or a medical history of Down syndrome [48].

#### Pemphigus vulgaris

A meta-analysis presented the association of *HLA-DRB1* polymorphisms with pemphigus vulgaris (PV). Especially DRB1*04, DRB1*08, and DRB1*14 were positively associated with PV, but DRB1*03, DRB1*07, and DRB1*15 were revealed to have a negative association [49].

A study from 2012 [50] suggested a co-existence of PV and HT, but the results were based on only a rather small study group, thus making a definitive interpretation of an association between PV and HT not possible. The study compared the rate of HT in 80 healthy patients to the rate of HT in 80 patients with PV. The following results were assessed: 9% of the PV patients were diagnosed with HT, compared to an only 1.2% presence of HT within the healthy control group [50].

### Rheumatology

#### Fibromyalgia

Fibromyalgia is a disease characterized by chronic widespread pain with changing locations in muscles and joints and a heightened pain response to pressure. Besides this, many patients also suffer from fatigue, sleep problems, and malfunction of memory—to mention only some of the heterogeneous symptoms going along with fibromyalgia [51].

In the current literature, no significant association between HLA genes and fibromyalgia could be found so far [52, 53].

To date, only few studies have investigated the correlation between fibromyalgia and HT. In order to provide a scientifically sound conclusion, more research will have to be done on this topic. Nevertheless, the existing studies seem to indicate a correlation between these two medical conditions.

Ahmad et al. [54] investigated the abovementioned association in 204 patients with established rheumatoid arthritis (RA). TgAb were tested positive in 24%, TPOAb in 29%. Of the autoantibody-positive patients, 40% suffered from fibromyalgia versus 17% of the autoantibody-negative patients (OR 4.641, *p* < 0.001). These results indicate an association between autoimmune thyroiditis and fibromyalgia in patients with RA [54]. Results of research by Bazzichi et al. [55] point in the same direction. Comorbid fibromyalgia resulted in 12 patients (31%) with HT and in 0% of patients with subclinical hypothyroidism. Based on their data, Bazzichi et al. suggested that the association between HT and fibromyalgia is more than just hypothetical [55].

#### Sjögren’s syndrome

The meta-analysis from Cruz-Tapias et al. presented that the alleles DQA1*05:01, DQB1*02:01, and DRB1*03:01 are high-risk factors for developing Sjögren’s syndrome (SS), whereas the alleles DQA1*02:01, DQA1*03:01, and DQB1*05:01 have protective effects [56]. A study from 2006 [57] investigated the co-occurrence of autoimmune thyroid diseases (AITD), in particular HT and Grave’s disease (GD), with other autoimmune diseases. It included two groups of patients. The first group consisted of 1517 patients with several autoimmune diseases including SS, and the second group included 426 patients with HT or GD. The evaluation of the first group showed that 7% of the patients had HT, whereas in the second group, HT patients showed in 17% a co-existence with SS [57]. Based on these results a correlation between HT and SS can be suspected. It has thus been proposed to screen patients with SS for HT and vice versa [57].

#### Systemic lupus erythematosus

Two meta-analyses, one from Europe and one from Latin America [58, 59], suggested an association with *HLA-DRB1* polymorphisms which can be boosted by combination with other genetic factors [59].

In a recent study of 301 patients with systemic lupus erythematosus (SLE), the prevalence of HT was 12.6% as compared to 5.6% in controls [60]. Disease activity and cumulative damage of SLE was not related to HT or to antibodies [60]. Another study evaluated Malaysian patients with SLE [61]. The frequency of AITD was assessed in 189 patients with SLE. AITD were found in 6.3%, and 3.7% had a thyroid disorder with hypothyroidism. In the study by Biró et al. [57] the prevalence of AITD was evaluated in 1517 patients with different systemic autoimmune diseases including SLE. According to the study, the prevalence of HT was 90-fold higher than in the general population. Through this increased risk, the authors conclude that screening for co-existing thyroid disorders is important in patients with systemic autoimmune diseases [57].

#### Systemic sclerosis

A study with 585 systemic sclerosis (SSc) patients and 458 controls of Chinese origin revealed an association with *DRB1* polymorphisms. The authors also mentioned that their results match with other studies of Spanish, US-Caucasian, and Hispanic populations [62].

In 2014 a study was published investigating the correlation between AITD and SSc. It included 210 patients with SSc, among whom 29 were diagnosed with HT (13.8%). Interestingly, these 29 patients were all females [63]. According to this study it seems to be useful to look for HT in patients with SSc [63].

### Endocrinology

#### Type 1 diabetes mellitus

Diabetes mellitus type 1 (T1DM) is caused by autoantibodies against insulin or glutamic decarboxylase, or both. Only in rare cases are antibodies directed against islet antigen‑2. An association between T1DM and thyroid autoimmunity is well studied, and the present literature clearly points out that there is a significant connection.

The majority of studies dealing with this topic point in the same direction. They represent the results of numerous other studies [64, 65]. Genetics is the primary risk factor for beta-cell autoimmunity. Although an environmental trigger is generally needed, T1DM mainly occurs in patients with either *HLA-DR3-DQ2* or *HLA-DR4-DQ8* haplotypes, or both [64].

A 12-year nationwide, population-based, retrospective cohort study by Lu et al. [66] pointed out that in children and adolescents with T1DM, the incidence of AITD was significantly higher as compared to those without T1DM [66].

Kahles et al. [65] evaluated the Type 1 Diabetes Mellitus Genetics Consortium Autoantibody Workshop data (7083 subjects), differentiating between T1DM patients with and without TPOAb or thyroid disease with respect to polymorphisms including HLA, also taking the ethnic origin into account. TPOAb were present in 25.2% and thyroid disease in 8.4%. Further associations were found with older age, female sex, and the presence of other autoantibodies. The highest prevalence was seen in patients with Hispanic ancestry (31%), the lowest in those with African ancestry (8%). The authors conclude that all patients (non-Hispanic whites, Hispanics, Asians, and Africans) with T1DM, especially those with advanced age and women, have thyroid pathologies to a high percentage, necessitating regular thyroid monitoring during follow-up investigations. Moreover, their study reveals that there might be a specific genetic contribution. But due to small numbers in Hispanic and African ancestry groups generating a low power, they refer to future studies investigating this issue [65].

In summary, the present literature clearly reveals a significant association between T1DM and thyroid autoimmunity. It is important for the primary care practitioner to be aware of these conditions and to screen for them, because early treatment of both conditions can lead in general to better disease control and improved health [67].

### Gastroenterology

#### Primary biliary cholangitis

Primary biliary cholangitis (PBC) is a chronic and slowly progressive liver disease accompanied by destruction of the bile ducts. It is considered to be an autoimmune disease. In the majority of cases it occurs in females between the fourth and the sixth decade of life.

A meta-analysis from Li et al. [68] presented that polymorphisms in HLA-DR*7 and HLA-DR*8 alleles on the one hand lead to a higher appearance of PBC, whereas on the other hand polymorphisms in HLA-DR*11, HLA-DR*12, HLA-DR*13, and HLA-DR*15 alleles seemed to have a protective effect [61].

Floreani et al. [69] investigated extrahepatic autoimmune conditions associated with PBC. From 361 PCB patients (339 females, 22 males), 61.2% had at least one extrahepatic autoimmune condition. Out of these, 45 patients (20.4%) had HT [69]. Mantaka et al. [70] showed that dyslipidemia, Raynaud syndrome, Sjögren syndrome, and HT were all significantly associated with PBC. Furthermore, even in first-degree relatives were these conditions significantly increased [70].

#### *Helicobacter pylori* infection

A European study by Kunstmann et al. [71] investigated the association between HLA class II genes and *Helicobacter pylori* (HP) infection. The *HLA-DRB1* locus was studied in 382 Germans with a positive *H. pylori* status, revealing no association between *HLA-DRB1* and HP infection [71].

A small clinical study considered an association between HT and HP. They compared the HP infection rate of 40 healthy individuals and 43 patients affected by HT. They calculated a statistically significant OR of 7.2, suggesting an association between HP and HT [72]. However, due to the small number of patients, this correlation has to be regarded questionable.

Two studies by Bassi et al. [73, 74] found a positive correlation between HP and Grave’s disease, but a lacking association with HT [73].

A meta-analysis by Shi et al. [75] analyzed seven studies involving a total of 862 patients. An association with AITD could be confirmed. For Grave’s disease a significant OR was found, but a non-significant OR of 1.45 (*p* = 0.11) for HT [75].

#### Autoimmune pancreatitis

The study by Ota M. et al. [76] investigated 43 patients with autoimmune pancreatitis (AIP) as compared to 213 healthy Japanese as controls. Two critical genes for the prevalence of AIP could be found: one region from HLA class II (HLA-DRB1*0405-DQB1*0401) and another from HLA class I (*ABCF1* proximal to C3-2-11, telomeric of *HLA‑E*). However, no literature which approves or denies an association with HT was found.

#### Celiac disease

Based on a meta-analysis from Diaz-Redondo et al. [77], an association between celiac disease and *HLA-DQ2/DQ8 *has been reported. In 302 patients with positive TPOAb, the prevalence of celiac disease was 1.3%, similar to that in the general population [78]. However, in another study [79], 16 out of 104 patients with HT (15%) were positive for celiac serology and 5 patients were diagnosed with celiac disease by endoscopy (4.8%). *HLA-DQ2* (and/or *HLA-DQ8*) was present in all these 5 patients and in 53 patients with HT (50%). Of 184 patients with celiac disease, 39 (21%) were positive for thyroid serology, 10 with euthyroidism (5%), 7 with subclinical hypothyroidism (3.8%), and 22 with overt hypothyroidism indicative of HT (12%) [79].

All in all, an association between celiac disease and HT can be assumed. Moreover, the presence of celiac disease was reported to be a risk factor for development of seronegative arthritis in patients with AITD [80].

#### Autoimmune atrophic gastritis

Autoimmune atrophic gastritis is caused by antibodies against parietal cells and intrinsic factor, which leads to mucosal destruction. Primarily the corpus and fundus of the stomach are affected, with the progression to a severe gastric atrophy eventually affecting the whole stomach [81, 82]. The clinical consequence is hypochlorhydria-dependent iron-deficient anemia, followed by pernicious anemia due to the lack of intrinsic factor necessary for vitamin B12 resorption [81]. Concerning the associations of pernicious anemia and iron deficiency with HT, also see the separate sections on pernicious anemia an iron deficiency below.

Autoimmune atrophic gastritis is frequently found in patients with thyroid diseases [81, 82]. Based on the literature, HT has been associated with gastric disorders in 10–40% of patients and, on the contrary, about 40% of patients with autoimmune gastritis also presented with HT [81]. In another study, patients with AITD had atrophic gastritis in about one third of cases [83]. Malabsorption of levothyroxine may occur in these patients as well [81]. This association has been also included in polyglandular autoimmune syndrome type IIIb [81]. Some similarities concerning the pathogenic mechanism of these two disorders have been found involving complex genetic, embryological, and immunologic interactions, as well as environmental factors [81]. Interestingly, celiac disease, another frequent autoimmune condition, seems to play a protective role for autoimmune atrophic gastritis [82].

### Other disorders

#### Pernicious anemia

A study from 1981 [84] showed an association between *HLA-DR* and pernicious anemia. It compared patients with pernicious anemia (*n* = 66) with healthy controls (*n* = 120). From these 66 patients, 18 had associated endocrine diseases. Differences were found between the amount of HLA-DR2, -DR3, -DR4, and DR5. The study concluded that aberration of these HLA antigens may result in a predisposition for pernicious anemia with and without endocrine diseases. HLA-DR3/4 may also predispose patients with pernicious anemia to endocrine diseases [84].

A small study [85] included 24 patients with different types of polyglandular autoimmune diseases (PGA): 2 with PGA1, 10 with PGA2, 10 with PGA3, and 2 without confirmed PGA. Pernicious anemia was present in 7 (2 with PGA2 and 5 with PGA3) of the 24 patients. Hypothyroidism was common in these patients, but without mentioning specific numbers [85]. Another study [86] evaluated the frequency of vitamin B12 deficiency in patients with AITD. Vitamin B12 levels of 115 patients (7 men, 108 women) with AITD were measured. Low B12 levels were found in 28% of the patients and fasting gastrin levels were elevated in 8 of 26 patients. Furthermore, in these 27 patients, parietal cell antibodies were positive in 8 out of 27 patients. According to this study the prevalence of pernicious anemia based on high serum gastrin levels in patients with low vitamin B12 levels was 31%. Consequently, the evaluation of B12 levels and further examination for pernicious anemia would make sense in patients with AITD [86].

#### Iron deficiency

Thyroid metabolism is impaired in case of iron deficiency, as thyroid peroxidase (TPO) is a heme (iron-containing) enzyme. TPO is responsible for the production of thyroid hormones. It is activated at the apical surface of thyrocytes only after binding heme. Iron deficiency is frequently present in patients with HT, as autoimmune gastritis is a common disorder associated with HT impairing iron absorption, (see section above). Treatment with iron could improve thyroid-hormone concentrations in anemic women with impaired thyroid function [87]. Noteworthily, therapy with both thyroxine and iron resulted in a more effective improvement of the iron status [87].

#### Vitamin D deficiency

Miettinen et al. [88] suggested an association of HLA polymorphisms with the concentration of 25-hydroxy (OH) vitamin D (25-OHD). They genotyped *HLA‑B* (*n* = 395), *HLA-DRB1* (*n* = 501), and *HLA-DQB1* (*n* = 475) of pregnant women and concluded that there is an association to serum concentrations of 25-OHD [88].

Clinically, vitamin D has been associated with the occurrence of autoimmune disorders and immune regulation [89]. Serum 25-OH vitamin D levels were inversely correlated with TPOAb and TgAb levels. Arslan et al. [90] could also show that TPOAb positivity was more common with severe and moderate vitamin D deficiency. In addition, a negative correlation between 25-OHD levels and TSH could be found [90]. Severe vitamin D deficiency, defined as 25-OHD < 10 ng/ml, was seen in 48.3% patients with HT and euthyroid function due to L‑thyroxine medication, in 35.0% of patients with newly diagnosed HT and euthyroid function, but in only 20.5% of euthyroid controls without HT. In a recent study [91] of 5320 individuals, patients with HT presented with lower 25-OHD as compared to non-HT patients. Comparable results were reported by Tamer et al. [92], with vitamin D insufficiency (defined as 25-OHD < 30 ng/ml) being present in 92% of HT patients and in only 63% of healthy controls. In a study [93] on children, the differences were even greater: 73.1% of patients with HT had vitamin D deficiency as compared to only 17.6% in controls, and TPOAb levels were inversely correlated with 25-OHD levels [93]. Vitamin D supplementation in primarily vitamin D-deficient HT patients caused a significant decrease of 20.3% in TPOAb levels [94]. Finally, a positive correlation was also found between thyroid volume and 25-OHD [95].

All in all, vitamin D deficiency is associated with HT [90–95] and it seems to modulate thyroid autoimmunity with an increase in TPOAb and TgAb levels [90, 93]. Thus, a potential role of vitamin D deficiency and its severity may play a role in the development and progression of HT [89, 90, 94]. Evaluation of vitamin D status in patients with HT seems to be recommendable.

#### Myasthenia gravis

The neuromuscular disease myasthenia gravis (MG) is, in most cases, caused by circulating antibodies that block nicotinic acetylcholine receptors at the postsynaptic neuromuscular junction. It leads to episodic muscle weakness and fatigue.

A study [96] within the northern Han Chinese population comparing 91 MG patients with 171 healthy controls presented a positive association between *HLA-DRB1* and MG patients [96]. A study of Norwegians with 369 MG patients and 651 healthy controls showed an association to HLA-DRB1*15:01 [97]. Three other studies investigating patient groups from Turkey, Tunisia, and Saudi Arabia also pointed out associations of HLA genes with MG [98–100].

Tamar et al. [101] looked for coexistent autoimmune diseases in 75 patients with MG and for characteristic autoantibodies that are associated with the most relevant forms of autoimmune diseases. 39 patients (52%) were autoantibody positive. The most frequent autoantibodies detected were thyroid autoantibodies and antinuclear antibodies. Yeh et al. [102] determined the risk of MG in patients with allergic or autoimmune thyroid disease in a national population-based case–control study in Taiwan. In this study, 1689 adult patients with MG were matched to those without MG by sex and age. Patients with HT showed a higher risk of MG (adjusted OR 2.87; range 1.18–6.97). The study also pointed out that the increasing severity of the thyroid or allergic disease was associated with an increase in the risk for MG [102]. In a retrospective data analysis by Meng [103], 106 patients with MG were reviewed. Clinical features including the relationship between thyroid function, antibodies, clinical course, and prognosis were investigated. The results showed that MG is often accompanied with thyroid pathologies and that patients with MG are more affected by HT and other AITD. However, no significant correlations between MG remission rate and TSH levels, total antibody levels, or TgAb and TmAb levels were seen in this study [103].

#### Glomerulonephritis

Several studies in the Chinese population observed a correlation with different HLA antigens for glomerulonephritis triggered by primary IgA nephropathy or anti-glomerular basement membrane disease. The results may predict a higher prevalence towards the disease and a worse prognosis for patients with those suspected genes [104–106].

However, so far there is no proof of a relationship between glomerulonephritis and HT. According to a retrospective study, there is the same HT prevalence among patients who have glomerular nephritis and those who are healthy [107].

## Discussion

Thyroid function may be normal or even slightly hyperthyroid at the beginning of HT, whereas in later stages of HT the thyroid function may remain either euthyroid or becomes hypothyroid. Sonographic features can vary depending on the phase and severity of HT. During the initial phase, the thyroid gland may be either normal in size or diffusely enlarged with a heterogeneous echotexture. Hypoechoic micronodules (1–6 mm) with surrounding echogenic septations are also considered to have a relatively high positive predictive value for HT. This sonographic pattern may be described as a pseudonodular or giraffe pattern. In later stages of HT the thyroid gland progressively decreases in size and the sonographic appearance of the thyroid gland finally shows a diffusely hypoechogenic pattern. Color Doppler studies usually show a normal or decreased flow, although in some cases, hypervascularity, not representing thyrotoxicosis, can be present. Besides sonography, the evaluation of thyroid antibodies is essential for diagnosis of HT. TgAb are found in approximately 70% and TPOAb in 90–95% of cases. A small portion of patients reveal negative thyroid antibodies, although sonography and the clinical presentation indicate HT (antibody-negative HT). All in all, it may be difficult to diagnose HT at an early stage of the disease, whereas the diagnosis of a clinically evident HT is usually an easy task, putting together the clinical presentation with signs of hypothyroidism, the hypoechogenic sonographic appearance, and usually positive thyroid antibodies. With the diagnosis of HT, these patients may need L‑thyroxine medication in case of hypothyroidism, whereas euthyroid HT patients will not need thyroid medication as long as they remain euthyroid, These HT patients will only require frequent sonographic follow-up controls due to the higher prevalence of PTC in HT.

**Fig. 2:**
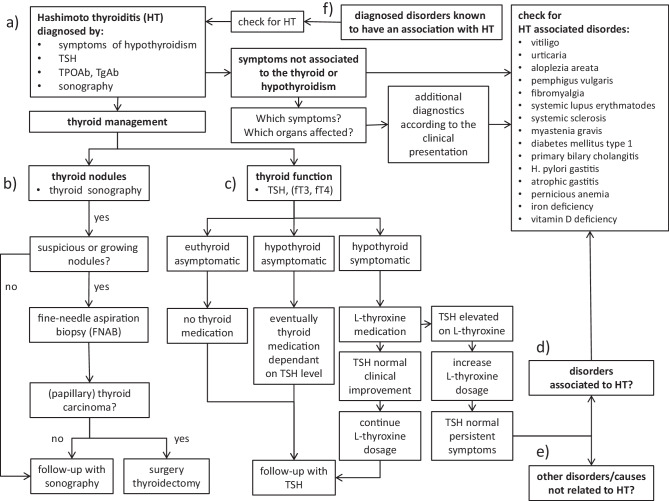
The workflow presents the different steps to diagnose Hashimoto thyroiditis (HT) by TSH, thyroid antibodies and sonography (**a**). The thyroid management of thyroid nodules with sonography and eventually fine-needle aspiration biopsy covers the evaluation of potentially malignant thyroid nodules aside HT (**b**). Thyroid function may need L‑thyroxine medication in case of hypothyroidism (**c**). Besides this, further diagnostics are warranted in all those cases with symptoms not related to hypothyroidism or the thyroid gland itself. In these cases, further evaluation should be performed, checking in particular for different disorders associated with HT (**d**) or other disorders not associated with HT (**e**). In case other autoimmune disorders are primarily diagnosed, an evaluation of coexisting HT should be included in the diagnostic work-up of these patients as well (**f**). *HT* Hashimoto thyroiditis, *TSH* thyroid stimulating hormone, *fT4* free L-thyroxine, *fT3* free triiodothyronine, *TPOAb* anti-thyroid peroxidase antibodies, *TgAb* thyroglobulin antibodies

With a diagnostic and therapeutic procedure focused only on the thyroid gland, most of the patients with HT can be diagnosed and managed sufficiently, ensuring normal thyroid function and surveillance of possibly growing thyroid nodules (Fig. 2, points a–c). However, a small proportion of patients with HT continue to complain about symptoms even after normalization of thyroid function. In these cases, diseases concomitantly occurring with HT, as presented above, should be considered (Fig. 2, point d) or even other disorders not associated with HT (Fig. 2, point e). The question is how to proceed in clinical practice in order to broadly evaluate possible concomitant disorders. At first, a detailed clinical and physical examination, not only focused on the thyroid gland, should be performed in these cases and, in particular, symptoms seen in HT-associated disorders should be checked (Fig. 2). By this, some disorders may be diagnosed at a glance, such as vitiligo or urticaria. Others will need further investigations including imaging (e.g., abdominal sonography, computed tomography, magnetic resonance imaging) and further specific laboratory parameters to diagnose these additional autoimmune disorders (Fig. 2). Furthermore, the coexistence of HT with other autoimmune disorders, as pointed out above, has also to be seen from another viewpoint. Those patients having autoimmune disorders with a known association to HT should also be screened for HT (Fig. 2, point f), as outlined by different authors in their conclusions [35–37, 44, 57, 63, 67, 86, 89, 90, 93]. In all of those disorders with a documented increased OR for coexistence with HT, a clinical evaluation for HT, even in case of any clinical symptoms indicative for HT, should be included into the diagnostic work-up of these disorders. This would include sonography of the thyroid gland and laboratory evaluation with TSH, TPOAb, and TgAb. By this, even the early subclinical presence of possible coexisting HT could be diagnosed, thus optimizing treatment of the affected patient. All in all, this evolves into a complex diagnostic work-up of patients with HT and possibly other autoimmune disorders and of patients with other autoimmune disorders which are known to show associations with HT, thus needing thyroid screening. Finally, iron (particularly in menstruating women) and vitamin D should be regularly evaluated in patients with HT to correct any deficiency, as thyroid function is dependent on iron and vitamin D status, as pointed out above [87, 90].

In certain cases, the HLA status may also be of interest. Evaluation of the HLA status in patients with HT and additional clinical symptoms not explained by HT itself could provide an approach to evaluate additional autoimmune disorders or a possibly increased risk for certain autoimmune disorders. The association of *HLA-DR* with HT and the published studies was rated according to the quality and quantity of data published (Tab. 1).

**Tab. 1:** The table shows the different diseases which were evaluated concerning an association with Hashimoto thyroiditis. The strength of association with Hashimoto thyroiditis and the presence of HLA II polymorphisms was graded based on the available literature

Disease	Association with Hashimoto Thyroiditis	HLA-Class II polymorphisms
Papillary thyroid carcinoma	+++	++
Vitiligo	+++	+++
Urticaria	+++	++*
Alopecia areata	++	+++
Pemphigus vulgaris	++	+++
Fibromyalgia	++	o
Sjogren‘s syndrom	++	+++
Systemic lupus erythematosus	++	+++
Systemic sclerosis	++	+++
Juvenile rheumatoid arthritis/rheumatoid arthritis	0	+++
Primary biliary cholangitis	++	+++
Helicobacter pylori infection	0/+	0
Autoimmune pancreatitis	0	++
Celiac diseases	0	++
Diabetes mellitus type 1	+++	++
Vitamin D deficiency	++	++
Pernicious anamia	+	++
Myasthenia gravis	++	+++

*0* studies which show no association with Hashimoto Thyroiditis, + only case reports or case studies or single center (*n* < 50), ++ single-center studies, +++ multicenter studies or meta-analysis or single-center studies (*n* > 1000), *Asterisk* studies available which deny an association with HLA

In case of certain HLA subtypes known to be more frequently associated with certain disorders, a closer surveillance of these patients may be additionally implemented in their follow-up controls.

In summary, this approach of a broader and multidisciplinary view on HT and associated autoimmune disorders would broaden and improve clinical management of patients with HT, with a better handling of their various complaints.
